# Isolated Monoclonal Human Urine-Derived Stem Cells Showed Differential Therapeutic Effects on Renal Ischemia–Reperfusion Injury in Mice

**DOI:** 10.3390/biomedicines13122911

**Published:** 2025-11-27

**Authors:** Guiyang Huo, Jie Geng, Xuanhe Liu, Guangrui Huang, Anlong Xu

**Affiliations:** School of Life Sciences, Beijing University of Chinese Medicine, Beijing 100029, China; h429827061@163.com (G.H.); gengjie@bucm.edu.cn (J.G.); liuxuanhe1998@163.com (X.L.)

**Keywords:** acute kidney injury (AKI), human urine-derived stem cells (hUSCs), monoclonal strain, transcriptome sequence, ischemia–reperfusion injury

## Abstract

**Objectives**: To investigate the characteristics of monoclonal human urine-derived stem cells (hUSCs) obtained through different culture protocols and compare their therapeutic effects on renal ischemia–reperfusion injury in mice. **Methods**: Monoclones of hUSCs derived from the urine of healthy volunteers were isolated and cultured using two different culture media. Flow cytometry, qRT-PCR and RNA sequencing were employed to characterize each monoclonal clone of multipotent stem cells across multiple passages. To evaluate their therapeutic effects on unilateral renal ischemia–reperfusion injury in BALB/c mice, 5 × 10^5^ hUSCs from each monoclonal clone were intravenously administered to mice via the tail vein, followed by assessments using Masson staining, qRT-PCR and renal tissue transcriptomics analysis. **Results**: Four monoclonal strains were successfully isolated from four fresh urine samples of a healthy young male volunteer: three cultured in EGM-MV medium and one in our modified medium. All four strains demonstrated stable expression of mesenchymal stem cell-related markers over eight passages of expansion. Bioinformatics analysis of multiple cell transcriptome datasets revealed that these four cell strains are more closely related to kidney tissue than to bone marrow mesenchymal stem cells (BMSCs), adipose-derived mesenchymal stem cells (ADMSCs), induced pluripotent stem cells (iPSCs), embryonic stem cells (ESCs), and urothelial cells. Additionally, significant differences were observed in the expression of genes associated with kidney development among the four monoclonal strains. Furthermore, the therapeutic effects of different monoclonal clones on renal ischemia–reperfusion injury in mice showed notable variability. **Conclusions**: The isolated monoclonal urine-derived stem cells in this study were showed closer transcriptomic similarity to renal progenitor cells than to other mesenchymal stem cell types and possessed differential therapeutic effects on acute kidney injury.

## 1. Introduction

In recent years, stem cell-based therapies have garnered increasing attention, emerging as a promising avenue for various clinical interventions [1,2]. The development of stem cell-derived organoid metabolic models, exosome [3,4], and other innovative therapeutic approaches has marked a significant stride toward commercialization [5,6]. However, the high costs of acquisition, the time required for cultivation, and associated ethical dilemmas have become pressing concerns in stem cell research [7,8].

Among various multipotent stem cells—such as those derived from bone marrow, adipose tissue, and amniotic fluid—human urine-derived stem cells (hUSCs) stand out for their robust proliferative capacity and the absence of ethical concerns due to their non-invasive collection method [9,10]. They hold significant potential for applications in reprogramming, genetic mechanism studies, and exosome research, where a substantial supply of human stem cells is essential. hUSCs also exhibit superior endothelial differentiation and angiogenic potential, along with increased secretion of growth factors, positioning them as strong candidates for vascular repair [11]. Notably, a 2024 study demonstrated that intravenous administration of 5 × 10^5^ human hUSCs significantly alleviated histological destruction and restored renal function in glycerol-induced acute kidney injury (AKI) mice, with therapeutic effects comparable to bone marrow-derived mesenchymal stem cells [12]. Additionally, the ease of collecting urine-derived stem cells significantly expands the donor pool. This feature is crucial for autologous therapies, as it can greatly improve patient compliance by avoiding the allogeneic immune response. Moreover, hUSCs can be induced to generate induced nephron progenitor cells (iNPCs) that reduce glomerular hypertrophy and tubulointerstitial fibrosis in diabetic nephropathy models, further highlighting their potential in treating chronic kidney diseases [13]. In summary, urine-derived stem cells represent an ideal, cost-effective option for personalized and precise cell therapies, offering a new approach for model establishment and drug screening platform construction [12,14,15,16].

Despite these advantages, variations in the morphology of hUSCs and uncertainties regarding their origin have been reported [17,18,19]. These variations may be attributed to the culture media used. Consequently, it is imperative to select an appropriate culture medium for hUSCs and establish monoclonal cell lines to clarify their tissue origin. This will facilitate more targeted and efficient research and practical applications. A key study using bioluminescence tomography imaging confirmed that hUSCs home to the tubules and glomeruli of injured kidneys within 3 h of injection in AKI mouse models, and their incorporation into damaged renal tissue was accompanied by reduced expression of the injury marker KIM–1, which provides direct evidence for the renal origin of hUSCs and their regenerative capacity [20].

Renal ischemia–reperfusion injury is a major cause of acute kidney injury and drives the progression of chronic kidney disease [21]. For patients with end-stage renal disease, kidney transplantation remains the optimal treatment. However, outcomes in managing ischemia–reperfusion injury before and after transplantation have not fully met the desired standards [22,23]. Stem cell therapy has emerged as a promising treatment for renal ischemia–reperfusion injury, leveraging its ability to home to injury sites, paracrine signaling, immune modulation, and inflammation reduction [24,25,26,27,28]. Recent mechanistic research has revealed that exosomes derived from hUSCs carry lncRNA TUG1, which interacts with the RNA-binding protein SRSF1 to regulate ACSL4-mediated ferroptosis, thereby mitigating renal ischemia–reperfusion injury [29]. This finding uncovers a novel molecular pathway underlying the therapeutic effects of hUSCs, enriching our understanding of their action mechanisms in renal repair.

Most stem cell therapies under current investigation use induced pluripotent stem cells or those derived from non-renal tissues. Studies have shown that differences in organ/tissue specificity lead to markedly varying therapeutic efficacies [30,31]. hUSCs have been identified as renal-derived and have demonstrated an innate capacity to migrate to injured renal tissue, as evidenced by previous research [32,33]. In human renal organoid models damaged by cisplatin, hUSCs not only integrated into the injured nephron structure but also reduced KIM–1 expression, confirming their regenerative potential in human–derived renal tissue models [20]. This characteristic endows hUSCs with enhanced potential for therapeutic intervention in renal ischemia–reperfusion injury [34]. Furthermore, hUSCs exert protective effects on kidneys by inhibiting oxidative stress and inflammation, which are key pathological processes in ischemia–reperfusion injury, making them more targeted than non–renal derived stem cells in renal repair [35].

However, it is recognized that distinct hUSCs, derived from different kidney tissues, may exert divergent therapeutic effects. In light of this, we established multiple monoclonal clones of hUSCs. These were then intravenously injected into mice with renal ischemia–reperfusion injury to evaluate their therapeutic capabilities. Our focus is to examine the origins of hUSCs cultured in different media and assess their distinct therapeutic impacts on ischemia–reperfusion injury.

## 2. Materials and Methods

### 2.1. Monoclonal Isolation and Expansion of hUSCs

Human urine-derived stem cells (hUSCs) were cultured using two distinct media: EGM-MV medium (CC-3125, Lonza Group Ltd., Basel, Switzerland), which has been previously reported in the literature [28], and a custom-modified medium developed in our study. The modified medium was composed of two components, Solution A and Solution B. Solution A was formulated with unsupplemented KSFM medium (10744019, GIBCO BRL, Grand Island, NY, USA) and supplemented with 5 ng/mL EGF (C029, Novoprotein Scientific, Inc., Shanghai, China), 50 ng/mL FGFb (C046, Novoprotein Scientific, Inc., Shanghai, China), and 1% PIS (15140148, GIBCO BRL, Grand Island, NY, USA). Solution B consisted of a 75% DMEM (11965092, GIBCO BRL, Grand Island, NY, USA) and 25% F-12K (21127022, GIBCO BRL, Grand Island, NY, USA) mixture, with additional supplements including 10% FBS (10099158, GIBCO BRL, Grand Island, NY, USA), 10% ITS (41400045, GIBCO BRL, Grand Island, NY, USA), 0.4 µg/mL hydrocortisone (HY-N0583, MedChem Express, Monmouth Junction, NJ, USA), 10 ng/mL EGF (C029, Novoprotein Scientific, Inc., Shanghai, China), 10 nM all-trans retinoic acid (atRA; R2500, Sigma-Aldrich, St. Louis, MO, USA), and 1% PIS (15140148, GIBCO BRL, Grand Island, NY, USA). The final hUSCs culture medium was prepared by mixing Solution A and Solution B at a 1:1 ratio. This study protocol was approved by the Ethics Review Committee of Beijing University of Chinese Medicine (approval No. BUCM-2023021006-1193) and conducted in compliance with the ethical guidelines outlined in the World Medical Association’s Helsinki Declaration. A total of four 200 mL urine samples were obtained from a healthy 25-year-old male donor. Each sample was aliquoted into 50 mL centrifuge tubes and centrifuged at 1000× *g* for 10 min within 30 min of collection. After discarding the supernatant, the urine precipitate was gently resuspended and washed twice with Dulbecco’s Phosphate-Buffered Saline (DPBS; 14190144, GIBCO BRL, Grand Island, NY, USA). The sediments from each urine sample were resuspended, collected in 1 mL of the corresponding medium, and inoculated onto separate cell culture dishes. Three of these samples were subjected to primary and subsequent culture using EGM-MV medium, yielding monoclonal clones designated as a, b, and c, respectively. The remaining one sample was cultured in the custom-modified medium (hUSCs culture medium) for both primary and subsequent passages, with the resulting monoclonal clone named d. After 24 h of culture, adherent cells were gently rinsed with DPBS to remove non-adherent cells and cellular debris. Primary cell growth was monitored using an IncuCyte imaging system. Well-formed clones derived from single cells with obvious expansion were selected, digested, and subcultured to obtain monoclonal hUSCs.

### 2.2. Identification Surface Markers of hUSCs Using Flow Cytometry

Cells were harvested and resuspended in 500 μL of staining buffer (phosphate-buffered saline, PBS, supplemented with 1% fetal bovine serum, FBS). The cell suspension was then incubated in the dark at 4 °C for 30 min to facilitate staining. Subsequent to incubation, cells were labeled with specific anti-human antibodies: Mouse Anti-Human CD44-FITC (MHCD4401, Invitrogen, Carlsbad, CA, USA), Mouse Anti-Human CD90-APC (17-0909-42, Invitrogen, Carlsbad, CA, USA), Mouse Anti-Human CD31-PE (MHCD3104, Invitrogen, Carlsbad, CA, USA), and Mouse Anti-Human CD34-PE-Cy7 (25-0349-42, Invitrogen, Carlsbad, CA, USA). Isotype control antibodies, including IgG-FITC (MG101, Invitrogen, Carlsbad, CA, USA), IgG-APC (MG105, Invitrogen, Carlsbad, CA, USA), IgG-PE (MG104, Invitrogen, Carlsbad, CA, USA), and IgG-PE-Cy7 (MG112, Invitrogen, Carlsbad, CA, USA) conjugates, were used to determine baseline fluorescence levels. The isotype controls and specific antibodies were separately incubated with cells in groups, under dark conditions at 4 °C for 30 min. After two washes with 3 mL PBS, cells were resuspended in 200 µL PBS and subjected to flow cytometry. Data were analyzed using a FACSCalibur™ analytical fluorescence-activated cell sorter (BD Biosciences, San Jose, CA, USA).

### 2.3. Quantitative Expression Analysis

The expression of marker genes was detected using 2× SYBR Green Pro Taq HS Premix (AG11701, Accurate Biology, Guangzhou, China). Quantitative reverse transcription-polymerase chain reaction (qRT-PCR) was performed to analyze gene expression profiles, with all primer sequences provided in the Supplementary Table S1. GAPDH was used as the endogenous reference gene for normalization. Total RNA (1 μg) isolated from individual hUSC colonies was reverse-transcribed into complementary DNA (cDNA) using the Evo M-MLV RT Kit with gDNA Clean for qPCR II (AG11711, Accurate Biology, Guangzhou, China) following the manufacturer’s instructions. The relative expression levels of target genes were calculated using the 2^−∆∆CT^ method. All qRT-PCR reactions and data analyses were conducted in a blinded manner, with the operator unaware of the sample group assignments to minimize experimental bias.

### 2.4. Animals

Six-week-old male BALB/c mice were purchased from Beijing Vital River Laboratory Animal Technology Co., Ltd., Beijing, China. Prior to experimentation, the mice were acclimatized for one week in a pathogen-free environment, with access to a standard rodent diet and water ad libitum. All animal-related procedures in this study were approved by the Ethics Review Committee of Beijing University of Chinese Medicine.

### 2.5. Renal Unilateral Ischemic Reperfusion Injury Model

All surgical procedures on mice were performed by the same researcher. Mice were randomly assigned to six groups (*n* = 6 per group): sham operation group, ischemia–reperfusion injury (IRI) group, and IRI groups injected with 5 × 10^5^ hUSCs derived from single colonies (a, b, c, d) using the random number expression method [36,37].

All animals were anesthetized with pentobarbitone (5 mg per 100 g body weight) and placed on a heating pad. For surgical preparation of the left renal region, hair was removed and the area was disinfected with iodophor. The dorsal skin and underlying tissues were dissected layer by layer, after which the left renal pedicle was exposed and clamped with a nontraumatic vascular clip. Following 40 min of clamping, the vascular clip was released, and the left kidney was gently repositioned into the abdominal cavity. The longitudinal incision on the left dorsal side was sutured layer by layer. Mice in the sham operation group underwent the same surgical procedures except for the application of the nontraumatic vascular clip.

At 12 h post-ischemia–reperfusion, monoclonal hUSCs were suspended in sterile physiological saline and administered to the corresponding mice via tail vein injection. Mice were euthanized at 48 h post-ischemia–reperfusion, and blood samples as well as organ tissues were collected for subsequent experiments.

### 2.6. Transcriptome Sequencing of hUSCs

Total RNA was extracted from monoclonal cell lines of groups a, b, c, and d. Transcriptome sequencing was performed on the Illumina NovaSeq 6000 platform by CapitalBio Corporation (Beijing, China). Raw sequencing data were quality-controlled using FastQC (v0.12.1). Clean reads were aligned to the human reference genome (GRCh38.p13) using STAR-aligner (v2.7.10b) with default parameters for RNA-seq mapping, including splice junction detection. Gene-level read counts were quantified using featureCounts (v2.0.6), a component of the Subread package.

Raw RNA sequencing data of nine cell lines—human adipose-derived mesenchymal stem cells (hADMSCs), human bone marrow-derived mesenchymal stem cells (hBMSCs), human embryonic stem cells (hESCs), human renal tubular epithelial cells (hHKCs), human induced pluripotent stem cells (hiPSCs), adult renal tubular progenitor cells (hKTPCs), human mesangial cells (hMESs), human podocytes (hPodcytes), and normal human urothelial cells (NHUCs)—were retrieved from The European Nucleotide Archive (ENA) database (relevant accession numbers are provided in Supplementary Table S2).

For normalization and differential expression analysis, all raw read counts (including our monoclonal cell data and ENA public data) were processed in R (v4.3.1) using DESeq2 (v1.40.1). Normalization was conducted via the median-of-ratios method to correct for inter-sample differences in sequencing depth. Differentially expressed genes (DEGs) were identified using thresholds of adjusted *p*-value < 0.05 and |log_2_ (fold change)| ≥ 0.5. Pathway enrichment analysis of these DEGs was performed using DAVID (v6.8) with a significance threshold of *p* < 0.05.

The raw RNA sequencing data generated in this study have been deposited in the NCBI Sequence Read Archive (SRA) under accession number PRJNA1169856.

### 2.7. Transcriptome Sequencing of Mouse Kidneys

Total RNA was extracted from left kidney tissues of the IR model group, sham group, and monoclonal cell lines (groups a, b, c, d). RNA integrity was assessed using the RNA Nano 6000 Assay Kit on the Agilent Bioanalyzer 2100 system (Agilent Technologies, Santa Clara, CA, USA). Library construction was performed with the qualified total RNA, and library quality was verified using the Agilent Bioanalyzer 2100 system. Libraries were sequenced on the Illumina NovaSeq platform by Novogene Co., Ltd., Beijing, China.

Raw sequencing data were quality-controlled using FastQC (v0.12.1). Clean reads were aligned to the mouse reference genome (GRCm39) using STAR-aligner (v2.7.10b) with default parameters for RNA-seq mapping (including splice junction detection). Gene-level read counts were quantified using featureCounts (v2.0.6), a component of the Subread package.

For normalization and differential expression analysis, raw read counts were processed in R (v4.3.1) using DESeq2 (v1.40.1). Normalization was conducted via the median-of-ratios method to correct for inter-sample differences in sequencing depth. Differentially expressed genes (DEGs) were identified using thresholds of FDR < 0.05 (adjusted *p*-value) and |log_2_ (fold change)| > 0.5. Functional enrichment analysis of DEGs was performed using DAVID (v6.8) with a significance threshold of *p* < 0.05.

The raw RNA sequencing data generated in this study have been deposited in the NCBI Sequence Read Archive (SRA) under accession number PRJNA1170919.

### 2.8. Masson’s Trichrome Staining and Quantitative Analysis of Renal Fibrosis

Kidney samples were fixed in 4% paraformaldehyde at 4 °C for 24 h, dehydrated through a graded ethanol series (70%, 80%, 95%, and 100%), cleared in xylene, and embedded in paraffin. Paraffin-embedded tissues were sectioned at a thickness of 4 µm using a rotary microtome (Leica, Wetzlar, Germany) and mounted on adhesive-coated glass slides. For Masson’s trichrome staining, collagen fibers were specifically stained blue with aniline blue, while muscle fibers and cytoplasmic components were stained red. Renal injury severity was semi-quantitatively scored by two independent researchers blinded to the experimental group assignments using a validated grading scale: Grade 0 (no detectable injury), Grade 1 (≤15% injured tissue area), Grade 2 (16–30% injured tissue area), Grade 3 (31–45% injured tissue area), Grade 4 (46–60% injured tissue area), and Grade 5 (>61% injured tissue area). Masson-stained sections were imaged at 200× magnification using a super-resolution microtissue imaging system (Leica, Germany); acquired images were processed with ImageScope x64 software (Version 12.4) and collagen-positive areas were quantified using ImageJ software (Fiji-win64, v2.9.0). For each sample, five randomly selected non-overlapping fields covering both cortical and medullary regions were analyzed to ensure representative sampling. Fibrotic area quantification was performed via standardized thresholding of collagen-positive regions using ImageJ software, and data were expressed as the percentage of fibrotic area relative to the total tissue area in each field.

### 2.9. Statistical Analysis

Data are expressed as mean ± standard deviation (SD). For comparisons across multiple groups, statistical analyses were performed using one-way analysis of variance (ANOVA), with subsequent Bonferroni post hoc tests to determine the significance of pairwise differences. All analyses were conducted with GraphPad Prism software (v9.4.1). A *p*-value < 0.05 was considered indicative of statistical significance.

## 3. Results

### 3.1. Culture and Characterization of Monoclonal Human Urine-Derived Stem Cells

**Figure 1:**
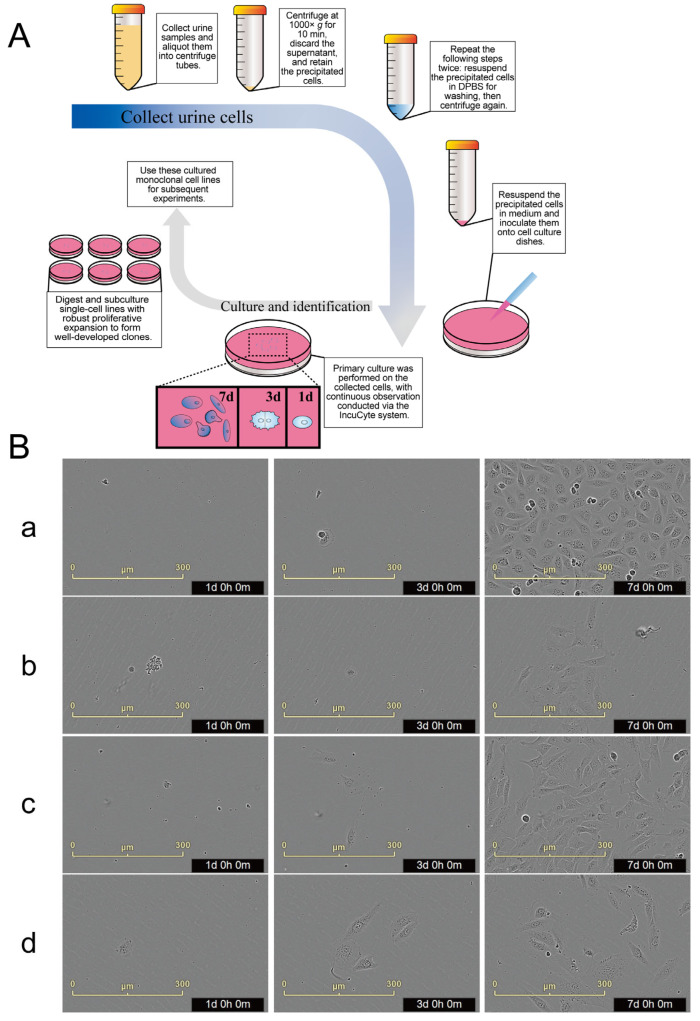
Establishment of monoclonal cell lines derived from human urine-derived stem cells. (**A**): Operation process of monoclonal isolation and culture of human urine-derived stem cells; (**B**): The proliferation and expansion process of monoclonal (**a**–**d**) human urine-derived stem cells (scale bar: 300 μm).

We collected fresh urine samples to isolate primary human urine-derived stem cells (hUSCs). Post-centrifugation, the precipitates were transferred into culture medium for the purpose to isolate monoclonal clones (Figure 1A). The cells’ growth was recorded using the IncuCyte imaging system (Figure 1B). We utilized two unique culture media for selecting stem cells: the standard EGM-MV and a custom medium optimized for our study. Clones derived from EGM-MV were named a, b, and c, whereas the one from our modified medium was called d. Every urine sample was collected from the same healthy donor to maintain consistency. The live cell tracking data revealed that adherent single cells were observed across all four hUSC monoclonal strains within the first 1–3 days post-primary culture, clear nuclei and expanded polygonal to rhomboid cytoplasm can be observed in clones a, b, c, and d. By the 7th day, these cells had proliferated sufficiently to form dense colonies (Figure 1B).

**Figure 2:**
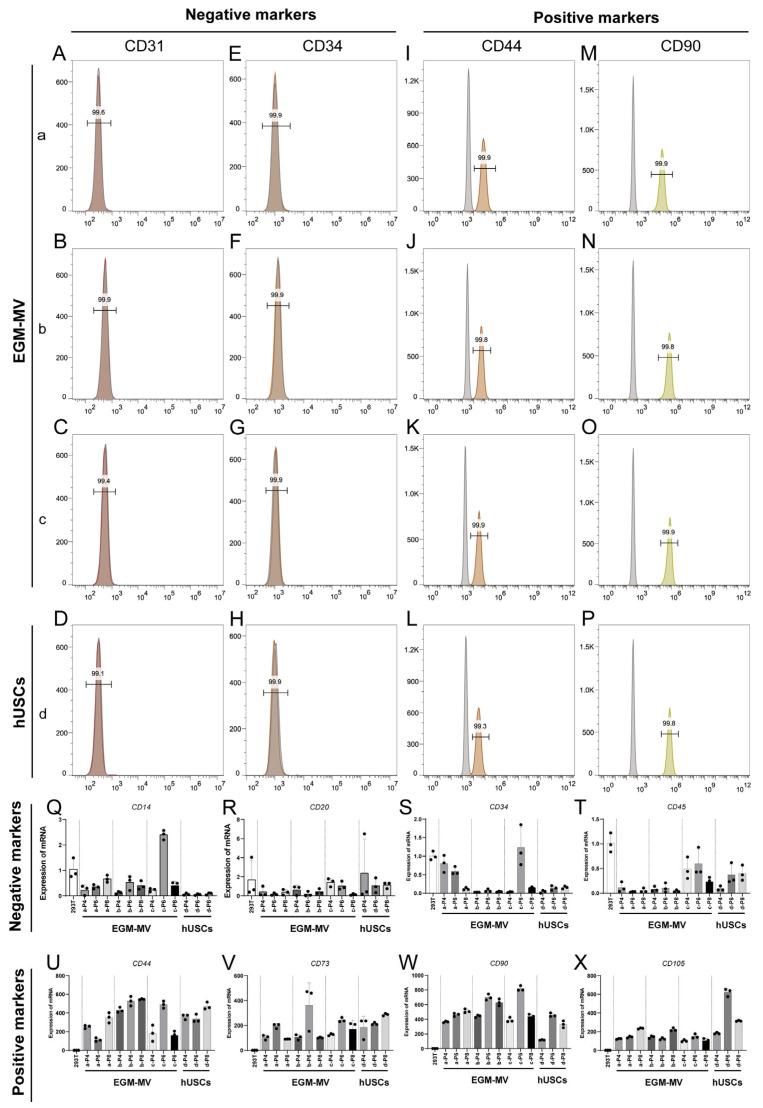
Identification of monoclonal human urine-derived stem cells. (**A**–**P**): Flow cytometry identification results of monoclonal (**a**–**d**) (P4). The ordinate represents the number of cells, and the abscissa denotes the relative intensity of signals in the corresponding fluorescence channel. The numbers above the bars indicate the proportion of cells with negative/positive expression of the corresponding antibody among the cells stained with the respective fluorescent antibody. The gray peak corresponds to the isotype control antibody group, while the other colored peaks represent the respective target antibody groups, negative expression of CD31 (**A**–**D**), negative expression of CD34 (**E**–**H**), positive expression of CD44 (**I**–**L**), positive expression of CD90 (**M**–**P**), data were compared with Isotype control antibodies group; (**Q**–**X**): qRT-PCR detection results of mesenchymal stem cell related genes (P4, P6 and P8), negative markers include *CD14* (**Q**), *CD20* (**R**), *CD34* (**S**), *CD45* (**T**), positive markers include *CD44* (**U**), *CD73* (**V**), *CD90* (**W**), *CD105* (**X**), data were compared with 293T cells group.

The flow cytometry analysis revealed that, at passage four (P4), all four monoclonal strains of cells did not exhibit the endothelial cell surface markers CD31 (Figure 2A–D) and CD34 (Figure 2E–F). Instead, they uniformly and strongly expressed the mesenchymal stem cell surface markers CD44 (Figure 2I–L) and CD90 (Figure 2M–P). The qRT-PCR data further confirmed the multipotent stem cell identity of these lines, showing low or undetectable levels of expression for the negative markers *CD14* (Figure 2Q), *CD20* (Figure 2R), *CD34* (Figure 2S), *CD45* (Figure 2T) across passages four, six, and eight (P4, P6, and P8), while the positive markers *CD44* (Figure 2U), *CD73* (Figure 2V), *CD90* (Figure 2W), and *CD105* (Figure 2X) were highly expressed.

### 3.2. Differentiating Renal Tissue Specificity Among Human Urine-Derived Stem Cell Monoclonal Clones

**Figure 3:**
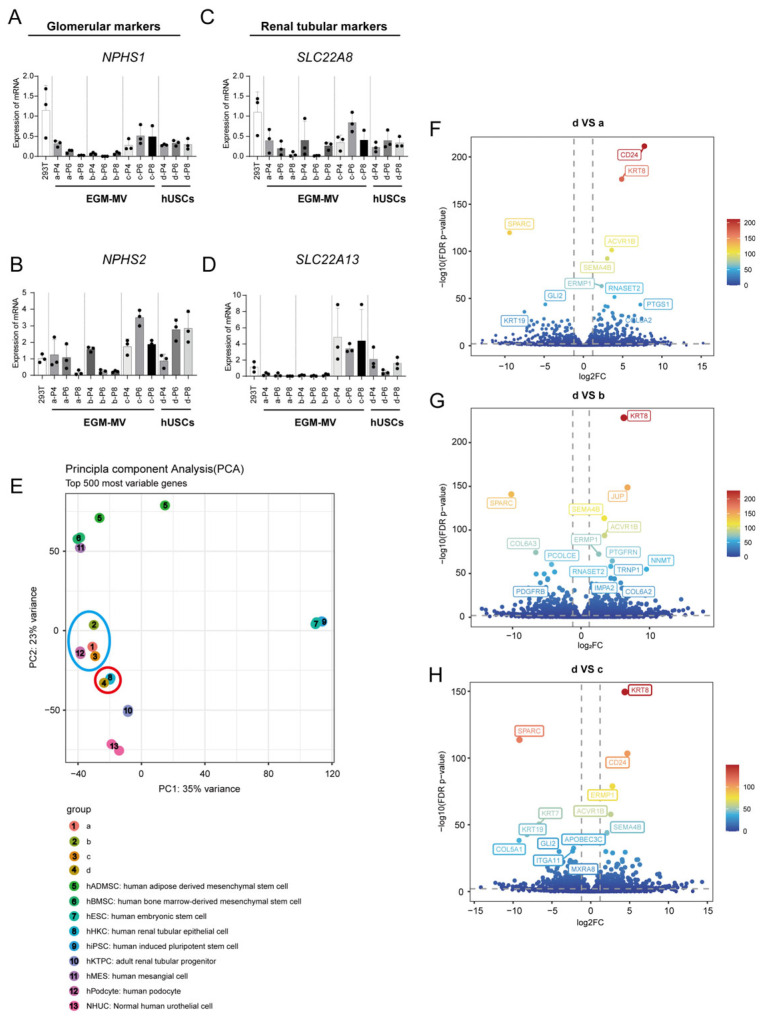
Cell identity of monoclonal human urine-derived stem cells. (**A**,**B**): qRT-PCR detection results of glomerular related genes, *NPHS1* (**A**), *NPHS2* (**B**); (**C**,**D**): qRT-PCR detection results of renal tubular related genes, *SLC22A8* (**C**), *SLC22A13* (**D**), data were compared with 293T cells group; (**E**): Transcriptome PCA results of various stem cells and human urine-derived stem cells (the red circle: monoclonal d and renal tubular epithelial cells; the blue circle: monoclonal a, b, c and glomerular podocytes); (**F**–**H**): Volcano plot of the DEGs of d vs. a (**F**), d vs. b (**G**), d vs. c (**H**), the upper right area divided by dashed lines indicated the significantly upregulated genes, The upper left area divided by dashed lines indicated the downregulated genes and other points indicated not significantly changed genes, respectively. The gray dashed horizontal line indicated the FDR  <  0.05, and the gray perpendicular dotted line indicated the |log2FC| > 0.5.

To explore potential differences in renal tissue specificity among the various monoclonal clones, we extracted RNA from clones a, b, c, and d for qRT-PCR and transcriptome sequencing analysis (Figure 3A–D). The qRT-PCR results indicated that at passages P4, P6, and P8, all four monoclonal lines exhibited negligible or low expression of mature glomerular podocytes marker genes *NPHS1* and *NPHS2* (Figure 3A,B), as well as mature proximal tubular cell marker genes *SLC22A8* and *SLC22A13* (Figure 3C,D). These findings suggest that these four human urine-derived stem cell monoclonal clones may lack the characteristics of mature glomerular or tubular cells.

In addition, raw RNA sequencing data of nine cell lines were retrieved from the public database The European Nucleotide Archive (ENA) (relevant accession numbers are provided in Supplementary Table S2). These public data, combined with our sequencing data, were subjected to principal component analysis (PCA) to assess the similarities between different cell types (Figure 3E). Consistent with previous studies [38], our PCA confirmed that all four hUSC clones clustered more closely with kidney-resident cells—including podocytes, renal tubular epithelial cells, and renal tubular progenitor cells—than with bone marrow-derived mesenchymal stem cells (BMSCs) or adipose-derived MSCs (ADMSCs), two widely utilized stem cell types in renal ischemia–reperfusion injury models (Figure 3E). This result validates the relevance of hUSCs as a cell source for renal research but does not represent a novel finding. This implies a higher likelihood that human urinary-derived stem cells have enhanced potential for renal differentiation and reparative capabilities. Notably, clone d showed a particularly strong correlation with renal tubular epithelial cells, suggesting a high degree of transcriptome gene expression similarity (Figure 3E, red circle mark), in the PCA’s dimension-reduced visualization, clone d was found to be closest to human renal tubular progenitor cells (Figure 3E), hinting at a renal repair capacity akin to that of tubular progenitor cells. Clones a, b, and c, derived from the EGM-MV medium, were relatively proximal to glomerular podocytes in the PCA mapping (Figure 3E, blue circle mark). Intriguingly, while clones a and c showed a close relationship with glomerular podocytes, clone b diverged from the other two, indicating transcriptome-level differences even among cells cultured from the same medium. A volcano plot analysis of the transcriptome data for differentially expressed genes (DEGs) revealed that the expression levels of genes related to tubular cell development and maturation, such as CD24, KRT8, and SEMA4B related to stem cell differentiation, were significantly upregulated in clone d compared to clones a, b, and c (Figure 3F–H), which also indicated that there were differences in kidney tissue specificity and cell differentiation related properties among different human urine-derived stem cell clones.

**Figure 4:**
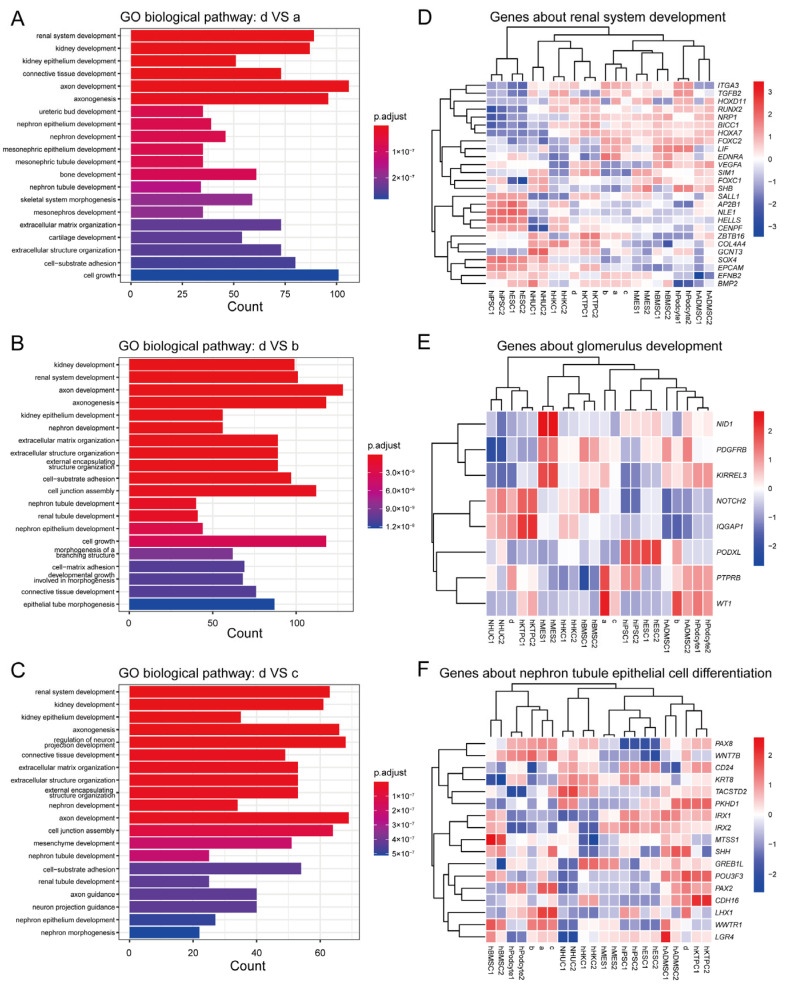
Differences in biological pathways and gene expression among monoclonal human urine-derived stem cells. (**A**–**C**): The top 20 of GO-biological process enrichment of the recovered DEGs profiles performed by DAVID online tool, d compare with a (**A**), d compare with b (**B**), d compare with c (**C**); (**D**–**F**): Cluster heatmap of DEGs (high expression, red; low expression, blue; The closer the branches cluster in the vertical gene dendrogram, the more similar the gene expression patterns are.), genes about renal system development (**D**), genes about glomerulus development (**E**), genes about nephron tubule epithelial cell differentiation (**F**).

In order to analyze the differential gene expression among the four groups of monoclonal cells and their possible renal origins, differential gene analysis and pathway enrichment analysis were performed (Figure 4A–F). The results showed that the differential genes between monoclonal d and monoclonal a, b, c were significantly enriched in kidney development-related pathways and tubular development-related pathways (Figure 4A–C), which is consistent with the results of PCA (Figure 3E). These results also indicate that the human urine-derived stem cells have different characteristics and differentiation potential due to different media, which may lead to a different effect of treatment in kidney related diseases. After normalizing all the transcriptome data used for PCA, the gene expression heatmap revealed that in the expression level of genes related to renal system development, monoclonal d was closer to renal tubular progenitor cells and renal tubular epithelial cells, while monoclonal a, b, and c were closer to renal mesangial cells and bone marrow mesenchymal stem cells (Figure 4D). In the expression level of genes related to glomerular development, monoclonal d was closer to renal tubular progenitor cells and urothelial cells, while monoclonal a and c were closer to podocytes, and monoclonal b was closer to adipose-derived mesenchymal stem cells (Figure 4E). In terms of the expression level of genes related to renal tubular epithelial cell differentiation, monoclonal d was similar to tubular progenitor cells, while monoclonal a, b, and c were more similar to podocytes (Figure 4F). This suggests that human urine-derived stem cells, despite originating from the same source, can exhibit distinct gene expression profiles under different culture media conditions. Even within the same medium, minor variations can arise between different monoclonal clones. Overall, at the transcriptome level, human urine-derived stem cells demonstrate a higher degree of kidney tissue specificity compared to other commonly used mesenchymal stem cells. This may imply that urine-derived stem cells cultured in different media may have different renal origins and therapeutic effects. However, this speculation requires further experimental verification.

### 3.3. Therapeutic Efficacy of Human Urine-Derived Stem Cells on Renal Ischemia–Reperfusion Injury in Mice

**Figure 5:**
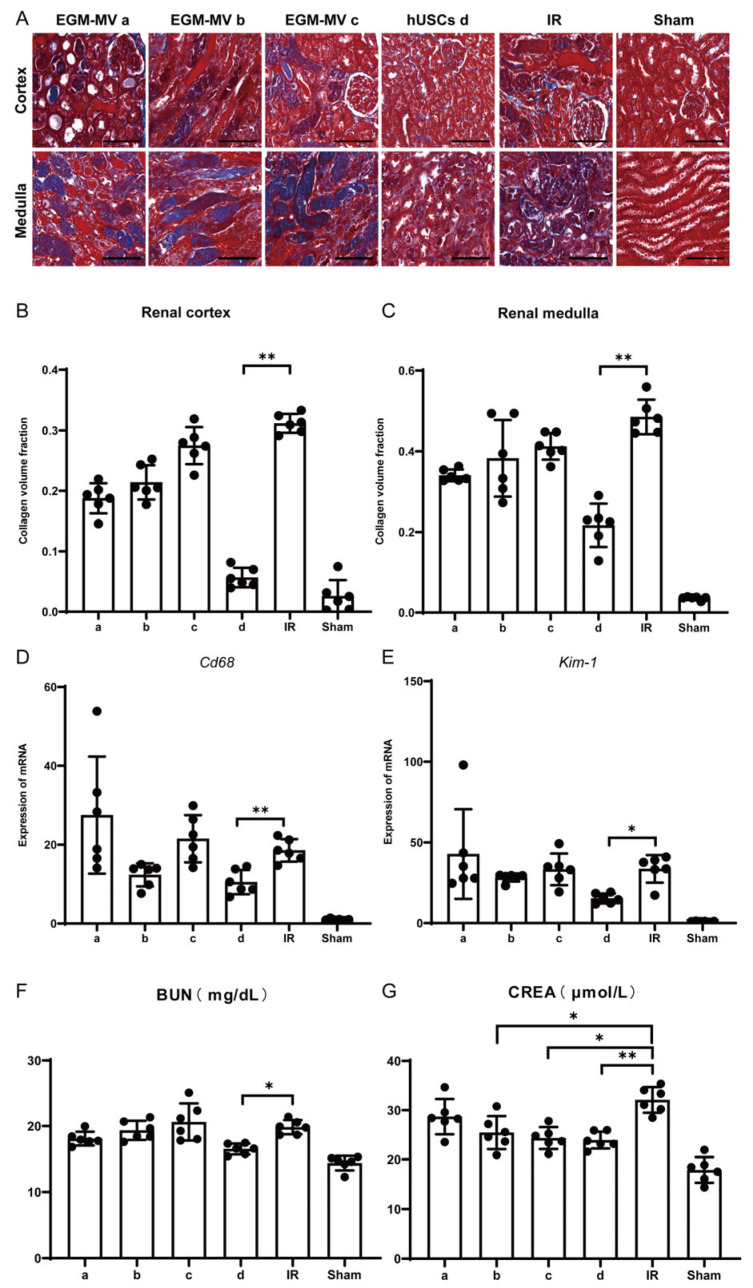
The effect of injecting monoclonal suspension of human urine-derived stem cells on renal ischemia–reperfusion injury in mice. (**A**): Representative images of left renal histopathological examination, Masson’s trichrome staining in EGM-MV a, EGM-EV b, EGM-MV c, hUSCs d, IR and Sham groups. Representative images were presented. Scale bar represents 100 μm. (**B**,**C**): Statistical analysis of collagen volume fraction, renal cortex (**B**), renal medulla (**C**); (**D**,**E**): qRT-PCR detection results of left kidney tissues, *Cd68* (**D**), *Kim-1* (**E**). Data were shown as means ± SD (*n* = 5, * *p* < 0.05, ** *p* < 0.01 vs. the IR group); (**F**,**G**): the serum biochemical results of BUN (mg/dL) and CREA (μmol/L) in mice.

To evaluate the therapeutic effects of different human urine-derived stem cell (hUSC) monoclonal clones on renal ischemia–reperfusion (I/R) injury, hUSC suspensions from individual clones were intravenously administered to mice at 40 min post-ischemia. Masson’s trichrome staining revealed that the monoclonal clone d-treated group exhibited reduced acute injury areas in both the renal cortex and medulla compared to the I/R group (Figure 5A), accompanied by a significant decrease in collagen volume fraction (Figure 5B,C). Further qRT-PCR analysis of inflammatory and kidney injury markers showed that the expression levels of *Cd68* (an inflammatory gene) and *Kim-1* (kidney injury molecule-1) in the clone d-treated group were significantly lower than those in the I/R group (*p* < 0.05; Figure 5D,E), indicating suppressed inflammatory responses. Additionally, serum biochemical analyses demonstrated improved renal function in the treated mice: serum urea nitrogen (BUN) levels were significantly reduced in the clone d-treated group compared to the I/R group (Figure 5F), while serum creatinine levels were significantly decreased in the clone b-, c-, and d-treated groups relative to the I/R group (Figure 5G). Collectively, these findings indicate that among the four monoclonal strains tested, clone d exerted superior efficacy in mitigating renal I/R injury, as evidenced by reduced tissue damage, suppressed inflammation, and improved renal function.

### 3.4. HUSC Cultured in Modified Culture Medium May Treat Renal Ischemia–Reperfusion Injury by Suppress Immune Response

**Figure 6:**
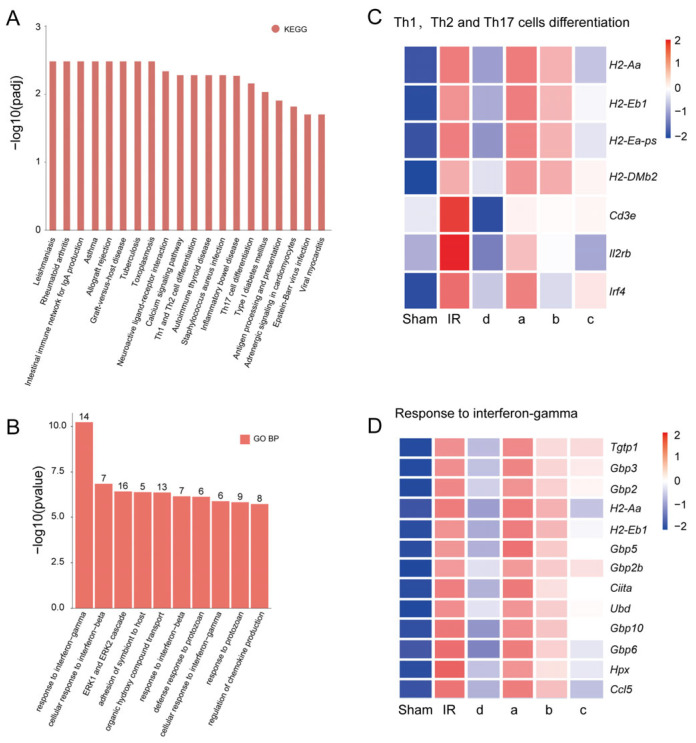
Transcriptome analysis of mice renal ischemia–reperfusion injury. (**A**): KEGG enrichment analysis on the differential metabolites of the monoclonal d group vs. IR group; (**B**): GO enrichment analysis on the differential biological progress of the monoclonal d group vs. IR group; (**C**): Cluster heatmap of genes about Th1, Th2 and Th17 cells differentiation; (**D**): Cluster heatmap of genes about pathway of response to interferon-gamma.

To investigate the possible mechanism of urine-derived stem cell monoclonal d in the treatment of renal ischemia–reperfusion, the RNA of left kidneys from all six groups was extracted for subsequent transcriptome sequencing analysis. The KEGG pathway analysis revealed significant alterations in the expression of genes associated with T-helper 1 (Th1) and T-helper 2 (Th2) pathways in the kidneys of mice that received the monoclonal d cell injection (Figure 6A). The gene expression heatmap demonstrated that the expression levels of genes related to Th17 cell differentiation were markedly reduced in the monoclonal d injection group compared to the I/R group and the other monoclonal injection groups (Figure 6C). In particular, regarding the CD3e gene, the key implication is that hUSCs may alleviate renal ischemia–reperfusion injury by regulating T cell-mediated adaptive immune inflammation. Additionally, the GO (Gene Ontology) analysis indicated a substantial decrease in the number of genes associated with the IFN-γ response in the kidneys of mice injected with monoclonal d, as opposed to the I/R group and other monoclonal treatment groups (Figure 6B). The heatmap of gene expression further illustrated that the expression levels of genes within the IFN-γ-related pathway in the monoclonal d injection group were significantly diminished relative to the I/R group and other monoclonal injection groups (Figure 6D). This pattern of expression more closely resembled that of the sham group. These results implying that the therapeutic mechanism of human urine-derived stem cells in renal I/R injury may be intricately linked to the suppression of local inflammatory reactions.

## 4. Discussion

Human urine-derived stem cells (hUSCs) have emerged as a promising candidate for multipotent stem cell-based research and applications, owing to their cost-effectiveness, noninvasive collection, and easy accessibility. These advantages make them valuable for personalized cell therapy, disease-specific pharmacological model development, and diagnostic purposes [34]. However, a critical challenge in the field is the lack of standardized culture protocols for hUSCs, with existing literature reporting the use of diverse culture media that can significantly alter the properties of cultured cells. Such variability may introduce inconsistencies in subsequent animal studies and translational research, highlighting the need for systematic evaluation of culture conditions and cell characteristics.

To address this gap, we cultured hUSC monoclonal clones using both the well-established EGM-MV medium and a custom-modified medium, and characterized their properties through flow cytometry and qRT-PCR. From passages 4 to 8 (P4–P8), all four monoclonal clones consistently expressed mesenchymal stem cell (MSC) hallmark markers (CD44, CD73, CD90, CD105) while lacking expression of endothelial cell markers (CD31, CD34) and hematopoietic/immune cell markers (CD14, CD20, CD45). This confirms that all clones meet the phenotypic criteria for MSCs and can serve as valid experimental models, though substantial differences among them were further revealed through subsequent analyses.

A long-standing debate in the field concerns the origin of hUSCs, which has not been fully resolved [18]. Our transcriptome bioinformatics analysis provides novel insights: hUSCs exhibit closer genetic similarity to kidney tissue cells (including renal tubular progenitor cells, renal tubular epithelial cells, and glomerular podocytes) than to urothelial cells or other commonly used MSCs (bone marrow-derived MSCs and adipose-derived MSCs). This transcriptomic proximity suggests a potential association with renal tissue lineage, but it is important to emphasize that this similarity does not constitute direct evidence of renal origin. Rather, it reflects functional and developmental relatedness that may underpin their renal repair potential. The research findings on hUSCs in the treatment of diabetic nephropathy (DN) further support this view, the study indicates that hUSCs can directly repair kidney tissue-specific cell damage by transferring functional mitochondria to damaged podocytes, which confirms the functional correlation between hUSCs and kidney tissue cells [39]. Consistent with previous studies [17,18], we observed significant gene expression differences among hUSC clones, which we hypothesize arise from two key factors: variations in culture media composition and intrinsic differences in the cellular sources within the original urine sample. Through a systematic comparison of the efficacy of hUSCs and bone marrow-derived MSCs in the treatment of acute kidney injury, it was found that both can improve renal function by inhibiting inflammation, reducing apoptosis, and promoting the proliferation of renal tubular epithelial cells [12]. Moreover, hUSCs have greater clinical translational potential due to their advantage of non-invasive acquisition. These findings underscore the necessity of establishing and characterizing hUSC monoclonal lines to reduce experimental variability caused by non-standardized culture protocols and heterogeneous cell populations.

Notably, our transcriptome analysis revealed distinct renal tissue specificity among the four clones. Clone d, derived from the modified medium, showed strong transcriptomic similarity to renal tubular progenitor cells and renal tubular epithelial cells, with significantly upregulated expression of genes related to tubular cell development and maturation (*CD24*, *KRT8*, *SEMA4B*) compared to clones a, b, and c (from EGM-MV medium). In contrast, clones a, b, and c clustered closer to glomerular podocytes in principal component analysis (PCA), though clone b diverged from a and c even under the same culture conditions, indicating inherent clonal heterogeneity. This finding is consistent with the results of studies on three-dimensional organoids derived from hUSCs in other research, the study revealed that kidney-specific extracellular matrix (kECM) can induce hUSCs to form organoids with renal functional characteristics, and there are significant differences in the tissue-specific differentiation tendencies of hUSCs under different induction conditions [40]. Pathway enrichment analysis further confirmed that differential genes between clone d and the other three clones were significantly enriched in kidney development and tubular development pathways, consistent with the PCA results. These differences in gene expression profiles likely contribute to the varying therapeutic efficacy observed in the renal ischemia–reperfusion (I/R) injury model. The therapeutic effects of hUSC monoclonal clones on renal I/R injury aligned with their transcriptomic characteristics. Among the four clones, clone d exhibited superior efficacy: Masson’s trichrome staining showed reduced acute injury areas in both renal cortex and medulla, with a significant decrease in collagen volume fraction. Molecular analyses and qRT-PCR analysis confirmed that clone d treatment significantly suppressed the expression of inflammatory marker *Cd68* and kidney injury marker *Kim-1*, while improving renal function as evidenced by reduced serum urea nitrogen (BUN) and creatinine (CREA) levels. This is consistent with the findings of a 2024 study on acute kidney injury (AKI). By establishing an AKI model in severe combined immunodeficient (SCID) mice via glycerol induction, the study confirmed that intravenous injection of human urine-derived stem cells (hUSCs) could rapidly restore renal function, reduce renal tubular apoptosis, and alleviate tissue damage [12]. Clones b and c also reduced serum CREA levels, but their overall therapeutic effects (including tissue protection and inflammation suppression) were less pronounced than clone d. These results highlight that the therapeutic potential of hUSCs is closely associated with their clonal characteristics, which are shaped by culture conditions and inherent genetic profiles. Regarding the potential therapeutic mechanism, our transcriptome analysis of mouse kidneys revealed that clone d treatment altered the expression of genes involved in T-helper 1 (Th1), T-helper 2 (Th2), and Th17 cell differentiation pathways, with a marked reduction in Th17-related gene expression compared to the I/R group and other clone treatment groups. Additionally, genes associated with the interferon-gamma (IFN-γ) response were significantly downregulated in the clone d group, resembling the expression pattern of the sham group. The latest mechanistic studies have further expanded this understanding: hUSCs can protect podocytes through a dual pathway, on one hand, they inhibit the PI3K/AKT/mTOR and ERK/mTOR signaling axes to activate autophagy; on the other hand, they transfer functional mitochondria to damaged podocytes via nanotube-like structures, directly repairing mitochondrial function [39]. Additionally, studies on acute kidney injury have confirmed that, similar to bone marrow-derived mesenchymal stem cells, hUSCs can exert paracrine therapeutic effects by secreting a variety of cytokines and growth factors, thereby promoting the proliferation of renal tubular cells and inhibiting their apoptosis [12]. These findings suggest that hUSCs may mitigate renal I/R injury by suppressing local inflammatory responses, particularly T cell-mediated adaptive immunity. However, it is important to note that these observations are based on transcriptomic changes, and direct evidence of immune cell modulation (e.g., alterations in immune cell infiltration or cytokine secretion) is lacking. Further functional validation is required to confirm this mechanistic hypothesis.

In conclusion, hUSCs represent a promising cell source for renal repair, but their clonal heterogeneity and culture-dependent properties necessitate rigorous characterization and standardization. Current studies have demonstrated that after being injected into mice, hUSCs are completely eliminated within 4–5 weeks without exhibiting toxicity or tumorigenicity, suggesting their potential for development as a cell therapy through optimized GMP-compliant procedures [41]. Our findings emphasize the importance of selecting appropriate hUSC clones and optimizing culture conditions to maximize therapeutic efficacy, laying the foundation for future preclinical and clinical studies on hUSC-based treatments for kidney diseases. However, the clinical translation of hUSC-based therapies requires comprehensive safety profiles and long-term outcome data, which remain to be established in future large-animal studies and controlled clinical trials.

## 5. Limitations

This study has several limitations that should be acknowledged. First, the conclusion regarding the potential renal association of hUSCs is based on transcriptomic similarity rather than direct evidence (e.g., lineage tracing experiments), and thus cannot definitively confirm renal origin. Second, the proposed immune regulatory mechanism relies on gene expression data, and additional functional analyses (such as CD3+ or F4/80+ immunohistochemistry to quantify immune cell infiltration, or ELISA to measure plasma cytokine levels) are needed to validate in vivo immune modulation. Third, while we measured key renal function markers (BUN and CREA), the inclusion of additional indicators (e.g., urinary protein excretion, glomerular filtration rate) would enhance the assessment of clinical relevance. Fourth, the heterogeneity among hUSC clones poses challenges for translational applications, as it highlights the need for standardized culture protocols, GMP-compliant production processes, and rigorous cell quality control measures to ensure consistent therapeutic efficacy. Fifth, all urine samples were collected from a single healthy donor, which may limit the generalizability of our findings; future studies should include multiple donors and diverse populations. Sixth, the present study focused on short-term therapeutic efficacy and did not evaluate the long-term safety or potential side effects of hUSC transplantation, such as immune reactions, ectopic tissue formation, or tumorigenic risk. These aspects are critical for clinical application and must be systematically addressed in future investigations. Seventh, the present study did not conduct a formal a priori sample size calculation. Instead, the sample size per group (*n* = 6) was determined based on precedents in the existing literature rather than rigorous statistical power analysis [34,35]. This approach may potentially compromise the study’s statistical power to detect subtle yet biologically meaningful effects, which should be considered when interpreting the generalizability of the findings. Finally, the discussion of hUSC-related research could be strengthened by integrating more recent literature on renal regeneration, including studies on exosome-mediated signaling pathways and progenitor cell identity, which would provide a more comprehensive contextual framework for our results.

## 6. Conclusions

In summary, our study successfully generated distinct monoclonal clones of human urine-derived stem cells (hUSCs) using two different culture media. Flow cytometry and qRT-PCR confirmed that these clones retained stable mesenchymal stem cell properties up to passage 8. Transcriptome analysis suggested that hUSCs are potentially associated with renal tissue lineage, with notable differences observed among the various monoclonal clones. Importantly, at the transcriptome level, hUSCs showed a closer similarity to renal progenitor cells compared to other commonly used mesenchymal stem cells, which reflects functional and developmental relatedness that may underpin their renal repair potential. Specifically, clones cultured in our modified medium exhibited a stronger resemblance to renal tubular progenitor cells than those grown in EGM-MV medium. Additionally, intravenous injection of hUSC monoclonal clones cultured in the modified medium exerted a significant anti-inflammatory effect in a mouse model of renal ischemia–reperfusion injury. These findings highlight the urgent need for further research into the stability, tissue specificity, inflammation-regulating capabilities, and underlying mechanisms of hUSCs, which may facilitate the development of a promising cell source for regenerative medicine and cell-based therapies.

## Data Availability

Data are contained within the article and Supplementary Materials, and materials and data supporting this study’s findings are available from the first or corresponding authors upon request. Raw RNA sequencing data have been deposited in the NCBI database under accession number PRJNA1170919.

## Supplementary Materials

The following supporting information can be downloaded at: https://www.mdpi.com/article/10.3390/biomedicines13122911/s1, Table S1: qRT-PCR primer sequence; Table S2: Transcriptome analysis referencing raw data.
