# Supplementary material for: Isolated Monoclonal Human Urine-Derived Stem Cells Showed Differential Therapeutic Effects on Renal Ischemia–Reperfusion Injury in Mice
Source: Biomedicines. 2025 Nov 27;13(12):2911. doi: 10.3390/biomedicines13122911 (PMC12730910; doi:10.3390/biomedicines13122911)
Supplement: Supplementary file 1 [file biomedicines-13-02911-s001.zip › biomedicines-3959147-supplementary.pdf]

Table S1：qRT-PCR primer sequence

Primer	Sequence (5'—3')
GAPDH-F	ACCCACTCCTCCACCTTTGAC
GAPDH-R	CTCTTCCTCTTGTGCTCTTGCTG
CD14-F	ACGCCAGAACCTTGTGAGC
CD14-R	GCATGGATCTCCACCTCTACTG
CD20-F	TGCACCCATCTGTGTGACTG
CD20-R	TTCCTGGAGTTTTTCTCCGTTG
CD34-F	CTACAACACCTAGTACCCTTGGA
CD34-R	GGTGAACACTGTGCTGATTACA
CD44-F	GACAAGTTTTGGTGGCACG
CD44-R	CACGTGGAATACACCTGCAA
CD45-F	TTCCTACAGACCCAGTTTCCC
CD45-R	AGGTGTTCGCTGTGATGGTG
CD73-F	GGCTGCTGTATTGCCCTTTG
CD73-R	GACTACTCTGTCTCCAGGTTTTCG
CD105-F	ACAGTACCCATACCCAAAACCG
CD105-R	CGATGAGGAAGGCACCAAAG
NPHS1-F	AGGACCGAGTCAGGAACGAAT
NPHS1-R	CTGTGAAACCTCGGGAATAAGACA
NPHS2-F	GCCCTGCCTGGATACCTACCACAA
NPHS2-R	TTCAGCCTCCACAGCCAGTGAGTG
SLC22A8-F	ATGGCCCAGTCTATCTTCATGG
SLC22A8-R	GACGGTGCTCAGGGTAATGC
SLC22A13-F	GAGGCGATACAACTGATCCAG
SLC22A13-R	TGTCCACAAACCAGACACAGA
Cd68-F	GTCTGATCTTGCTAGGACCGCTTAT
Cd68-R	GGGCTGGCTGTGCTTTCTG
Kim-1-F	TATCAGAAGAGCAGTCGGTAC

Kim-1-R	TGTTGAAATAGTCGTGGGTC

Table S2：Transcriptome analysis referencing raw data

Cell type	Raw data
ADMSC1	SRR12782290
ADMSC2	SRR12782290
hiPSC1	SRR19635647
hiPSC2	SRR19635648
hESCs1	SRR10207427
hESCs2	SRR10207428
hPodcyte1	SRR16200952
hPodcyte2	SRR16200953
hMES1	SRR16200958
hMES2	SRR16200959
hHKC1	SRR19909850
hHKC2	SRR19909851
NHUC1	SRR14509898
NHUC2	SRR14509899
hKTPC1	SRR7782620
hKTPC2	SRR7782621
hBMSC1	SRR22560355
hBMSC2	SRR22560357
